# Case report: Diagnosis and treatment of delayed epidural pyogenic abscess after brain tumor operation: a report of 5 cases and review of the literature

**DOI:** 10.3389/fsurg.2023.1202387

**Published:** 2023-06-28

**Authors:** HePing Shen, YingCong Lin, ZhengMin Chu, GengHuan Wang, WenLai Chu

**Affiliations:** ^1^ Department of Neurosurgery, Second Affiliated Hospital, Jiaxing University,, Jiaxing, China; ^2^ Department of Neurosurgery, Zhejiang Chinese Medical University, Hangzhou, China; ^3^ Department of Neurosurgery, Jiaxing Maternity and Child Health Care Hospital, Jiaxing, China

**Keywords:** brain tumor, surgery, epidural abscess, treatment, prognosis

## Abstract

**Objective:**

To explore the clinical manifestations and treatment of delayed epidural pyogenic abscess after brain tumor surgery.

**Method:**

To retrospectively analyze the medical records of 5 patients with delayed epidural pyogenic abscess after brain tumor surgery in our hospital from January 2010 to December 2020, including clinical manifestations, laboratory results, imaging findings, treatment measures, prognosis, etc. The causes of epidural abscesses were analyzed, and the treatment methods and prognosis were evaluated.

**Result:**

Among the 5 cases, there were 4 male and 1 female patient, aged 52–75 years. Three cases were gliomas and 2 cases were meningiomas. Four cases received postoperative radiotherapy, and 1 case had open frontal sinus during operation. None of the surgical incisions were infected. The time between the tumor surgery and the discovery of an epidural abscess was 1.5 to 24 months. All 5 patients had headaches, 1 case had a fever, and 2 cases had limb dysfunction. Three cases had elevated blood inflammatory markers. MRI- DWI showed restricted diffusion. All 5 patients underwent surgery, 4 patients had bone flap removed, and 1 patient had bone flap retained. Bacterial culture was positive in 3 cases and negative in 2 cases. All 5 cases were cured, followed up for 1.5–9 years, and no epidural abscess recurred.

**Conclusion:**

The clinical manifestations and laboratory results of delayed epidural pyogenic abscess after brain tumor surgery are not specific, but MRI-DWI has specificity. Postoperative radiotherapy for brain tumors and intraoperative opening of the frontal sinus may be associated with delayed epidural pyogenic abscess. For patients with normal skin flap and no serious inflammation of the bone flap, clinicians can attempt to preserve the bone flap.

## Introduction

The incidence of epidural pyogenic abscess has decreased markedly during the era of antibiotic therapy. Epidural pyogenic abscesses after brain tumor surgery are rare in clinical practice, and most are secondary to acute incision infection. Delayed epidural pyogenic abscesses are even rarer. Delayed epidural pyogenic abscess after surgery is a central nervous system focal infection that occurs more than one month after surgery with epidural abscess accumulation and no other surgical site infection. The surgical incision is uninfected, the infectious etiology remains unclear, and treatment options vary. In this article, we review the medical records of 5 patients with delayed epidural pyogenic abscesses secondary to brain tumor surgery in our hospital from the past 10 years and discuss the related clinical manifestations, laboratory results, imaging manifestations, treatment measures, and outcomes.

## Materials and methods

1. General information: Our study included 4 male and 1 female patient, aged 52–75 years. Inclusion criteria: (1) all cases were secondary to brain tumor surgery; (2) all epidural pyogenic abscesses appeared 1 month after surgery; (3) epidural pyogenic abscesses were confirmed by surgery. Exclusion criteria: (1) patients developed postoperative incision infection, intracranial infections including meningitis, subdural abscess, and brain abscess; (2) the patient developed an epidural abscess within one month after surgery (Table 1).
2. Preoperative preparations: Determine the patient's tumor type, whether the bone flaps were connected to the paranasal sinus, whether there are implants, and whether radiotherapy was performed after surgery. Analyze the clinical manifestations, laboratory examinations, and imaging findings of extradural pyogenic abscess.
3. Treatment measures: Before surgery, appropriate antibiotics were used. All 5 patients underwent epidural abscess debridement, and whether to retain the bone flap was determined according to the situation during the operation. Pus samples were collected intraoperatively for bacterial culture. After the surgery, all patients received systemic anti-inflammatory treatment.

**Table 1:** Clinical data for 5 cases of delayed epidural pyogenic abscess after brain tumor operation.

Patient no.	Sex/age(year)	Types and sites of surgery	implant material	frontal sinus opening	Radiotherapy	Clinical manifestations	Time from tumor surgery to diagnosis(Month)	Bone flap preservation	pus culture
1	M/68	Right parieto-occipital atypical meningioma	Yes	No	Yes	Headache,unilateral limb weakness	4	Yes	Staphylococcus epidermidis
2	F/75	Left temporoparietal meningioma	Yes	No	No	Headache	1.5	No	Negative
3	M/60	Left frontal diffuse astrocytoma	Yes	Yes	Yes	Headache,fever	24	No	Pseudomonas aeruginosa
4	M/63	Right parietal lobe diffuse astrocytoma	Yes	No	Yes	Headache,unilateral limb weakness	5	No	Negative
5	M/52	Right frontotemporal glioblastoma	Yes	No	Yes	Headache	2.5	No	Staphylococcus aureus

## Results

**Figure 1:**
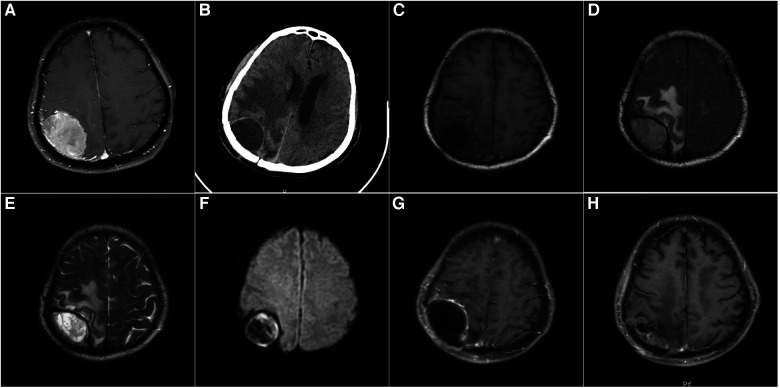
(**A**): A 68-year-old male patient with contrast-enhanced MRI: right apical meningioma, who underwent craniotomy for tumor resection. Postoperative pathology revealed atypical meningioma, and postoperative radiotherapy was performed. (**B**): Four months after the operation, the patient's left limb was numb and weak, without fever, and the skin flap showed no inflammation. CT showed a cystic lesion in the right parietal lobe. (**C**) T1-weighted MRI showed a hypointense lesion in the right parietal lobe. (**D**): FLAIR sequence of MRI showed a hyperintense lesion in the right parietal lobe. (**E**): T2-weighted MRI showed a hyperintense lesion in the right parietal lobe. (**F**) Diffusion-weighted MRI (DWI) showed a hyperintense lesion in the right parietal lobe. (**G**): Enhanced MRI showed a cystic lesion in the right parietal lobe with enhancement of the cyst wall. (**H**): The epidural abscess was removed, and the bone flap was preserved. After 5 months of surgery, MRI reexamination showed no recurrence of epidural abscess.

**Figure 2:**
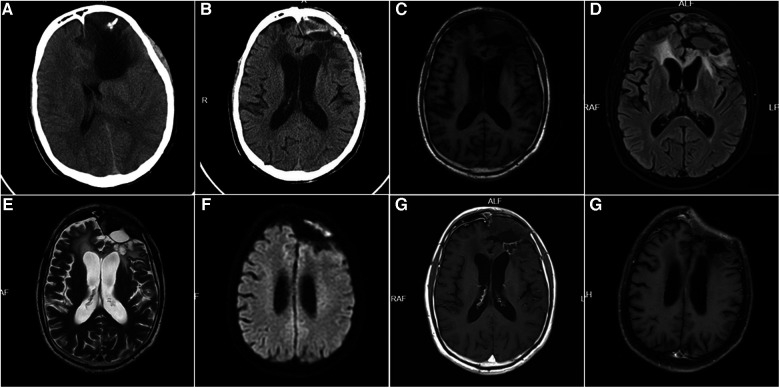
(**A**): A 60-year-old male patient with CT: left frontal glioma who underwent craniotomy for tumor resection. Postoperative pathology revealed diffuse astrocytoma and postoperative radiotherapy was performed. (**B**): 24 months after surgery, the patient had a headache accompanied by fever, and the skin flap showed no inflammation. CT showed the left frontal epidural lesion. (**C**): T1-weighted MRI showed an epidural hypointense and isointense lesion in the left frontal lobe. (**D**): FLAIR sequence of MRI showed epidural mixed signal lesions in the left frontal lobe. (**E**): T2-weighted MRI showed an epidural mixed signal lesion in the left frontal lobe. (**F**): Diffusion-weighted MRI (DWI) showed partial restricted diffusion in the left frontal epidural lesion. (**G**): Enhanced MRI showed an epidural lesion in the left frontal lobe with mild enhancement of the cyst wall. (**H**): The epidural abscess and the bone flap were removed. After 3 years of surgery, enhanced MRI reexamination showed no recurrence of epidural abscess.

1. Types and sites of surgery: 1 case of right parieto-occipital atypical meningioma, 1 case of left temporoparietal meningioma, 1 case of left frontal diffuse astrocytoma (intraoperative frontal sinus opening), 1 case of right parietal lobe diffuse astrocytoma and 1 case of right frontotemporal glioblastoma. All 5 patients had implants, including artificial meninges and titanium connectors. Three cases of glioma and one case of atypical meningioma underwent radiotherapy.
2. Clinical manifestations: The time from brain tumor surgery to the discovery of epidural abscess ranged between 1.5–24 months. All 5 patients had headaches, 1 with fever, and 4 without fever. No patient experienced vomiting or altered consciousness. Neurological examination revealed that two patients had limb movement disorders. Meningeal irritation signs were negative in 5 patients. All patients did not present with flap inflammation.
3. Laboratory results and imaging findings: 3 patients had elevated blood inflammatory indicators. Two patients underwent Lumbar puncture examination. The cerebrospinal fluid test results were normal. Computed Tomography (CT) examination showed hypodense lesions under the bone flap and outside dura matter. Magnetic resonance imaging (MRI) showed low signals on T1, high signals on T2 and FLAIR, restricted diffusion on DWI, and most patients had heterogeneous ring enhancement.
4. Time from brain tumor surgery to the discovery of epidural abscess: 1.5–3 months in 2 patients, 3–6 months in 2 patients, and 24 months in 1 patient.
5. Treatment measures: 3 patients with high inflammatory indicators were treated with antibiotics preoperatively. The other 2 patients showed no inflammation manifestations and were not treated with antibiotics before surgery. Epidural abscess debridement was performed in all 5 patients, with removal of bone flaps in 4 patients, and retention in 1 patient (Figure 1 and Figure 2). Three of the five patients were positive in pus culture, including 1 case of *Pseudomonas aeruginosa*, 1 case of *Staphylococcus epidermidis*, and 1 case of *Staphylococcus aureus*. For the remaining 2 patients, their pus cultures were negative. All patients received systemic anti-inflammatory therapy postoperatively. During 1.5–9 years of follow-up, no recurrence of epidural abscess was found.

## Discussion

Epidural abscess ranks third among all focal infections of the central nervous system, only after cerebral abscess and subdural abscess. However, it seldom occurs in clinical practice, which is a rare form of pyogenic infection, accounting for about 2% of all central nervous system infections (1). Moreover, most epidural abscesses in the central nervous system occur within the spinal canal and rarely occur in the intracranial region. There have only been sporadic reports of intracranial epidural abscesses in the literature (2).

Intracranial epidural abscesses are often caused by the direct spread of adjacent infections, among which rhinosinusitis, otitis media, mastoiditis, or orbital inflammation accounts for approximately 60%–90% (3, 4). Most intracranial epidural abscesses occur in children, adolescents, and young adults, with over 70% of patients being younger than 20 years (5). Other less common causes include scalp trauma and vascular dissemination (1). Another important clinical cause is direct dissemination following neurosurgery (1).

With the increasing frequency and complexity of Neurosurgery operations over the decades, the risk of post-operative central nervous system infections has become higher and higher, including epidural abscesses. Intracranial epidural abscesses are often a sequela of cranial osteomyelitis caused by incision infection (5, 6), which are also an extremely rare complication after craniotomy (7). According to statistical analysis by Hlavin et al., the incidence of epidural abscess after neurosurgery in three hospitals was 0.15% (7). Postoperative incision infection developed into osteomyelitis, which led to epidural abscess, and even meningitis or brain abscess. This complication usually occurs in the early postoperative period. However, Hlavin et al. found that the mean time from surgery to diagnosis of epidural abscess was 32.6 months (7). In the present study, the time from brain tumor surgery to the discovery of epidural abscess ranged between 1.5–24 months.

Clinical features of postoperative epidural abscesses appear to be completely different from those of patients without surgery (7). Hlavin et al. considered that most postoperative epidural abscesses were secondary to postoperative incision infections (7). However, all 5 patients in the present study had no flap inflammation. In neurosurgery, implant materials are often used, including artificial meninges, titanium connectors, and titanium meshes. Intracranial foreign bodies accompany an increased risk of infection (7). All 5 patients in this study had implants, including artificial meninges and connectors. Epidural abscesses are more likely to occur in patients who have undergone two or more surgeries, or in patients with postoperative cerebrospinal fluid leakage (7). The population with the highest risk of developing epidural abscesses after surgery is the age group of 50–60 years old, who are older than patients who have not undergone surgery(7). In the present study, all patients were over 50 years of age. Immunosuppression, alcohol, and drug abuse are major risk factors in non-surgical patients (8), but are rare among surgical patients. For surgical patients, intraoperative frontal sinus opening and postoperative radiotherapy are major risk factors. In this study, 1 patient had intraoperative frontal sinus opening and 4 patients received radiotherapy, which may be factors leading to epidural abscess. In Hlavin et al.'s report, although meningiomas accounted only for 15% of intracranial tumors, 50% of tumor surgeries involving infection were meningiomas, which was related to the relatively long surgical time for meningiomas and the widespread use of artificial meninges (7). Unlike cerebral abscess and subdural empyema, the clinical manifestations of epidural abscess are usually nonspecific (mainly headache and fever), and focal neurological signs are uncommon (7, 9). Epidural abscesses had been reported to cause depression and suicidal ideation (10). Hlavin et al. summarized the clinical features of patients with epidural abscess after craniotomy as follows: older age, less fever, wound infection, false-negative CT scan, and pathogenic gram-negative aerobic bacteria or skin flora (7).

CT scan is the easiest and most convenient technique for diagnosing intracranial diseases. Mittal et al. considered that contrast-enhanced CT was often the first choice since it was faster and easier to obtain, and was more advantageous in skeletal anatomy (11). However, in 30% of patients, CT scans alone cannot diagnose (7). CT may only reveal subtle findings, and sometimes it was difficult to determine whether the infection was epidural or subdural, whether the lesion was infectious, hemorrhagic, or cerebrospinal fluid (12). By contrast, MRI is more sensitive and can more accurately display the severity of infection, distinguishing whether an infection is epidural or subdural (1, 11). Abscesses are characterized by hypointensity on T1-weighted images and hyperintensity on T2-weighted images, with limited diffusion. Therefore, MRI is the preferred imaging technique for patients suspected of epidural abscess. However, the specificity and sensitivity of DWI for diagnosing intracranial infection are somewhat affected by blood composition after surgery (10). The absence of diffusion restriction on MRI DWI does not completely rule out the diagnosis of abscess, because the effect of paramagnetic susceptibility at the base of the skull near the craniofacial sinuses may affect signal scattering and create artifacts (13). Some scholars have found that Nuclear medicine is considered as an alternative to traditional MRI when evaluating the infection near the skull base, especially the application of new contrast enhancement agents in neuroradiology, and Nuclear medicine can improve the accuracy of postoperative infection detection (13, 14). Earlier literature reported that aerobic Gram-negative bacteria accounted for less than 10% of isolates, while in Hlavin et al.'s report, the aerobic Gram-negative bacteria were cultured in 52% of patients (7). There have also been reports of fungal cultures in the literature (15, 16), and mixed bacterial infections are common as well. Among our 5 patients, 3 cases had positive bacterial cultures, with 1 case for *Pseudomonas aeruginosa* and 2 cases for *Staphylococci*.

Antibiotic therapy is essential (1). Depending on the response to therapy, intravenous broad-spectrum antibiotic therapy should be continued for 2–4 weeks, followed by oral or intravenous supplementation for up to 8 weeks (5). In the presence of osteomyelitis, antibiotic therapy was recommended to be extended for a further 8 weeks or longer (1). Premature discontinuation of broad-spectrum antibiotics during treatment may result in insufficient infection control (12).

The necessity for surgery in some patients with epidural abscesses has been questioned (7). In the opinion of some scholars, patients with the following conditions may not need surgical treatment: (1) No neurological deficit; (2) No mass effect on CT scan; and (3) Antibiotics are effective. Some scholars believed that for patients whose lesions detected by MRI were too small for surgical drainage, careful imaging follow-up could be performed with antibiotics alone. However, most scholars believe that antibiotic therapy alone cannot block the infection progression, which is almost impossible to eradicate infection (7, 12).

For epidural abscesses not secondary to craniotomy, burr hole drainage may be considered (5). However, for epidural abscesses secondary to craniotomy, most scholars adopt craniotomy. Hlavin et al. reported that all patients underwent craniotomy and removed the bone flap (7). Currently, it remains controversial whether bone flap should be preserved. In my opinion, in some patients who did not have obvious osteomyelitis and did not need reoperation or radiotherapy, the bone flap can be preserved. In this study, bone flap was preserved in 1 patient, who had no recurrence of infection during 1.5 year follow-up. Since the dura mater can act as a mechanical barrier to prevent the pus from directly affecting the cortical vasculature and cerebral parenchyma, the prognosis is relatively good. Moreover, with improvements in diagnosis, antibiotics, and surgical interventions, the mortality has dropped significantly. Previously, the mortality rates were as high as 30%–50%, whereas recent reports described rates of 4%–10% (12, 17). Nonetheless, if the epidural abscess is not treated promptly, the dural barrier is likely to be breached by infection, which will pass through a vascular channel or erode the dura mater, resulting in subdural or cerebral abscess (12). For these patients, the prognosis depends on the successful treatment of other purulent processes. All 5 patients in this study had good outcomes.

## Conclusion

Intracranial delayed epidural pyogenic abscesses after brain tumor surgery are easily overlooked because of the absence of inflammation at the surgical incision. Moreover, clinical manifestations and laboratory findings are also nonspecific. MRI is very helpful for diagnosis, especially MRI-DWI, which has certain specificity. Postoperative radiotherapy for brain tumors and intraoperative opening of the frontal sinus may be associated with delayed epidural pyogenic abscess. For delayed epidural pyogenic abscess patients with normal skin flap and no serious inflammation of the bone flap, clinicians can attempt to preserve the bone flap.

## Data Availability

The original contributions presented in the study are included in the article/**Supplementary Material**, further inquiries can be directed to the corresponding authors.
